# Hands‐free from inoculation to harvest: Microbial fermentation with multivariate model to automate induction of recombinant protein expression

**DOI:** 10.1002/btpr.70055

**Published:** 2025-08-05

**Authors:** Jennifer Reid, Andrew Szto, Airong Chen, Patricia Gomes, Craig Kearse, Joyce Ni, Tao Yuan

**Affiliations:** ^1^ Global Bioprocess Development – Drug Substance Development Sanofi Vaccines Toronto Ontario Canada

## Abstract

Industrial fermentation continually improves biological process control for a wide range of microorganisms used in multi‐billion‐dollar industries including industrial enzymes, pharmaceuticals, foods, beverages, commodity chemicals, and bioenergy. In the case of recombinant protein production, batch and fed‐batch phases of fermentation are usually followed by an induction phase, where chemical or thermal induction initiates the expression of a target protein. Fed‐batch processes are usually automated, whereas “out‐of‐the‐box” distributed control systems (DCS) are often unable to define the threshold for induction and respond accordingly. The present study demonstrates the integration of optical density (OD) process analytical technology (PAT) and Lucullus®, a process information management system (PIMS), to enable end‐to‐end automated fermentation at bench and pilot scale. Data aggregated from tens of fermenter runs and hundreds of offline training measurements enabled the development of an accurate multivariate model to predict OD in real‐time. This eliminated the requirement to generate offline correlation models for each OD probe, allowed for model transfer, and incorporated additional predictor terms such as antifoam usage. Automating the induction phase enabled end‐to‐end fermentation, reducing labor and operational costs while increasing yield through higher reactor utilization within the same time period.

## 1 INTRODUCTION

Multi‐billion‐dollar industries rely on fermentation to deliver biological process control for a wide range of microorganisms, including bacteria (e.g., *Escherichia coli*), algae, yeast, and molds.^1^ These industries include the production of industrial enzymes (used in detergent, pulp and paper, textile, leather products, etc.), pharmaceuticals, dairy and foods, beverages, commodity chemicals, and bioenergy.^1^, ^2^, ^3^ Within the pharmaceutical sector, fermentation is essential for vaccine production, generating multiple products such as recombinant proteins, plasmid DNA, and adjuvant components.^4^, ^5^ Fermentation provides microorganisms with a near‐optimal environment to grow efficiently and produce the desired products. A well‐controlled fermentation process offers numerous advantages, including precise regulation of growth profiles and cost‐effective production.

There are multiple sequential stages in fermentation processes common to these applications, such as a “batch phase” followed by a “fed‐batch phase” to maintain nutrient levels and support exponential growth. The fed‐batch phase is typically automated using predetermined feed profiles,^6^ dynamic feedback control based on dissolved oxygen (DO) or pH,^7^ or adaptive control and/or in‐line process analytical technologies (PAT).^5^, ^8^ Additional automation can help maintain an optimal environment in the fermenter by controlling pH, dissolved oxygen (DO), and temperature setpoints.^9^

Many industrial applications require an “induction phase” to trigger the production of a desired target once sufficient biomass has been generated.^10^ While the fed‐batch phase is usually automated, the induction phase often is not, as the threshold of “sufficient biomass” can vary, and standard “out of the box” installations of distributed control system (DCS) software are typically not equipped to determine and act upon this information independently. Therefore, process changes may be required to align key fermentation decisions with daytime hours to allow manual intervention to start the induction phase. This limits production efficiency, raises operating costs, and can even lead to loss of data capture during overnight processes.^11^ Induction is typically triggered by adding a chemical agent (i.e., isopropyl β‐d‐1‐thiogalactopyranoside (IPTG), l‐arabinose, nalidixic acid, anhydrotetracycline) to bind a promoter or repressor element,^10^, ^12^ or through thermal induction; where an increase (range: 37–42°C)^13^ or decrease (range: 10–25°C)^12^ in fermenter temperature can trigger temperature‐inducible expression.

In the absence of automation, the threshold of sufficient biomass to start the induction phase is typically identified using manual sampling of the bioreactor and offline use of a spectrophotometer.^14^ However, fermentation ideally remains a closed system to avoid contamination by opportunistic organisms that can reduce yield,^3^ and to minimize potential operator exposure to hazardous chemicals and organisms. PAT, such as inline turbidity and optical density (OD) probes, enable real‐time biomass detection and reduce sampling needs.^2^, ^4^ Without this type of PAT, intensification strategies may suffer from lack of data capture during overnight activities, as noted in some reports.^11^ Unfortunately, initiating the induction phase via an automated response to inline OD appears to be rare, and existing reports could not be found at the time of this study.

Automating the induction phase offers numerous benefits, enabling end‐to‐end automated fermentation without operator intervention. These benefits include reduced labor and operation costs, improved product consistency, increased space–time yield, and comprehensive continuous data capture. The present study demonstrates the integration of PAT and process information management system (PIMS) software to enable end‐to‐end automated fermentation without operator intervention from inoculation to harvest, at both bench and pilot scale. While other vendor‐agnostic products and solutions could likely achieve similar results, we describe an automation solution using Dencytee (Hamilton) OD probes integrated into a Lucullus (Securecell) PIMS solution. Furthermore, data aggregated from tens of fermenter runs enabled an accurate multivariate model to predict OD in real time, resulting in multiple benefits: capturing additional predictor terms beyond the OD probe's capabilities, eliminating the need to generate new offline correlation models for each OD probe, enabling model transfer to new users, and allowing dynamic switching between models within a single run if required. Altogether, this approach allows fermenters to enter the induction phase overnight and be harvested in the morning, minimizing workload and maximizing process efficiency for a fully automated *E. coli* fermentation process producing recombinant proteins of interest.

## 2 METHODS AND MATERIALS

### 2.1 Microbial strain

*Escherichia coli* BL21(DE3) transformed with an IPTG‐inducible plasmid expressing a recombinant protein was used in this study. Five different recombinant proteins ranging from 30 to 110 kDa were assessed, as they were part of ongoing development projects. All recombinant proteins included were soluble and non‐toxic to the microbial host.

### 2.2 Offline measurements

Offline optical density (OD_600_) was measured at a fixed wavelength of 600 nm using a BioMate 160 UV–Vis spectrophotometer (ThermoFisher Scientific) with 0.9% saline as the blank and diluent.

### 2.3 SDS‐PAGE

Reducing sodium dodecyl sulfate‐polyacrylamide gel electrophoresis (SDS‐PAGE) was performed using NuPAGE® pre‐cast 10% or 4–12% Bis‐Tris gels, using 1× MOPS buffer as SDS‐PAGE running buffer, NuPAGE® LDS Sample Buffer, and NuPAGE® Sample Reducing Agent (all from Thermofisher Scientific). Samples were heated up to 70°C for 10 min, then 10–20 μL were loaded onto the gel and proteins were separated using the Xcell SureLock Mini‐Cell system (Invitrogen), in reducing conditions. Gels were stained with InstantBlue or ReadyBlue Protein Gel Stain (both from Sigma‐Aldrich) and de‐stained with ultra‐pure water. Gel images were acquired on a GS900 Densitometer scanner and processed with ImageLab (both from BioRad).

### 2.4 Fermentation conditions

Fermenters were inoculated from a seed flask cultured using standard conditions previously described.^6^ All cultivations used chemically defined, antibiotic‐free media. All chemicals met or exceeded U.S. Pharmacopeia and/or European Pharmacopeia quality standards. Fermentation was performed in 2‐L double‐walled glass Biostat B‐DCU (Sartorius) and 20‐L stainless steel Biostat Cplus (Sartorius) stirred‐tank reactors equipped with Rushton impellers and baffles. In brief, fermentation was operated in batch mode until the initial supply of glucose was exhausted, and a characteristic rise in dissolved oxygen (DO) triggered a carbon‐limited fed‐batch phase with the addition of a glucose‐containing nutrient feed solution at a specific growth rate.^6^, ^15^ One‐sided pH control with ammonium hydroxide was used, and antifoam (polyether‐ and oil‐based) was added as indicated in figure panels. Gas was supplied at a volumetric flow rate that ranged from one to two volumes of gas per volume of culture media per minute (1–2 vvm). The temperature was controlled by a water jacket at the desired setpoint. Protein expression was induced with IPTG when the culture reached the desired OD_600_ range in the bioreactor. Setpoint control was managed by the digital control unit of the fermenter (i.e., pH, DO, and temperature setpoint maintenance). An OD_600_ target was chosen for the decision criteria for induction threshold, instead of process duration, for several reasons. An OD_600_ target provides a consistent window to initiate the induction stage while allowing for potential changes in operating parameters of preceding stages. These changes–planned and unplanned–in the prior operating conditions include: inoculation (cell density, and any unexpected lag in growth), feed rate, and temperature parameters (both affecting growth rate and therefore process duration), and different yield requirements for various projects.

### 2.5 PAT and Lucullus

Process control, automation, process data centralization, visualization, and management were accomplished with Lucullus® (Securecell, version 24.1), referred to as a “PIMS” in this study. Lucullus was networked to the fermenters, external pumps, and the “Arc Modbus OPC Converter” (Hamilton Co.) to transmit continuous real‐time data from the Dencytee probes (Hamilton Co.) to Lucullus via open platform communications (OPC). The Dencytee probes are referred to as “OD probes” interchangeably in this study. An offline correlation model using the supplied ArcAir software (Hamilton Co.) was not developed. Instead, real‐time inline OD predictions were calculated using a multivariate equation developed in Dataiku and programmed into Lucullus with the same model features (more in Section 2.6). For automated induction, the PIMS was programmed to wait for the OD prediction to exceed a predefined OD target for induction. Once exceeded, a peristaltic pump was activated to withdraw a sample from the fermenter before induction (BI) and store it at 2–8°C until offline OD_600_ measurements could be made. Once sampled, another peristaltic pump was activated to add a pre‐determined volume of IPTG (kept at 2–8°C until use) for induction. The BI samples were used to confirm that induction was automated at the desired target OD and to validate the OD model predictions (explained in greater detail in Figure 3a). The PIMS was also used to store inline and offline OD measurements and control nutrient feed addition.

### 2.6 Statistics

Data preparation and iterative model building were completed using Dataiku (version 13.2.1). Ordinary least squares regression (OLSR) and Ridge regression were performed to create a multivariate equation that could be easily implemented in Lucullus to predict OD_600_ in real time during subsequent fermentation runs. Because of the dynamic bioreactor environment where gas bubbles can interfere with measurements, the raw transmission and reflection data were smoothed using an unweighted moving average with a 0.2 h window. Model building used *N* = 15 independent 2‐L fermentation runs with a combined total of *N* = 159 offline OD measurements taken during these fermentation runs. The offline OD dataset was randomly split into 80% training and 20% testing sets. The final model with sufficient accuracy used three latent variables with significant contributions to overall variance, in descending order of variable importance of prediction (VIP): transmission (data transformation: moving average, inverse), reflection (data transformation: moving average), and antifoam addition (data transformation: percent of fermenter volume). Additional model parameters and statistics are presented in Figure 2.

## 3 RESULTS

The OD probe, supplied with ArcAir software, is configured with four default offline correlation models. The software supports the creation of a custom offline correlation model for each independent probe, which requires an initial fermentation run to create a model with up to 6 offline correlation points over a desired dynamic range so that the probe can output a density value that can only be used during a subsequent fermentation run. This is required for every new probe and cannot be transferred from one probe to another (at the time of this study). Therefore, it is desirable to have a single model that can accommodate both old and new biomass probes.

**FIGURE 1:**
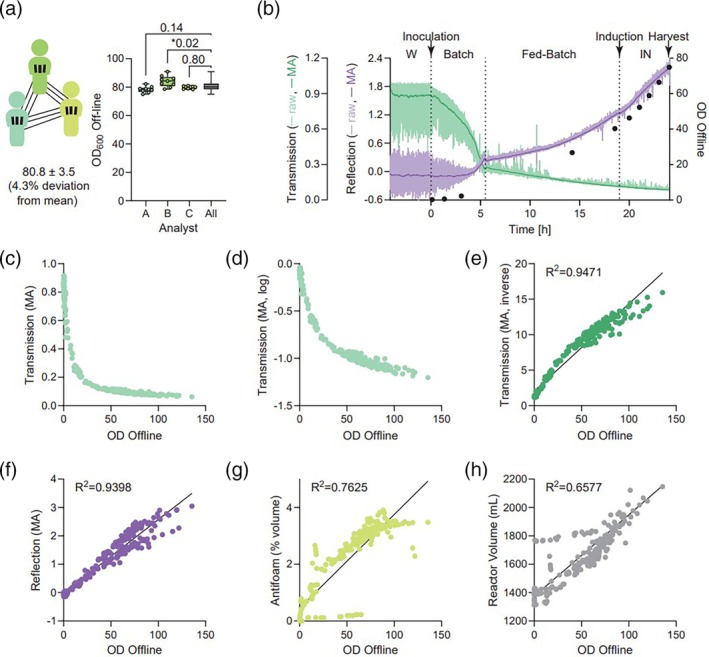
Visualization of inline OD probe data relative to offline OD. (**a**) Diagram depicting three operators each creating three samples and measuring all nine samples blind to the results. One‐way ANOVA, P values shown on plot. (**b**) Representative raw and moving average (MA) continuous data from OD probe collected during waiting (W), batch, fed‐batch, and induction (IN) phases. OD offline included for comparison. (**c‐h**) Biplots visualizing the relationship between transmission MA (**c**) log‐transformed transmission MA (**d**), inverse‐transformed transmission MA (**e**), reflection MA (**f**), antifoam as a percent of total fermenter volume (%vol) (**g**), and total fermenter volume (**h**). For biplots where a linear relationship was observed, linear regression was conducted, and the *R* ^2^ value is shown.

Prior to creating an inline OD model, the variability in offline measurements was first considered to set a minimum expectation of model accuracy. Three operators each collected three samples from a fermenter at the harvest stage, requiring multiple dilutions to measure within the linear range of the spectrophotometer (Figure 1a). Each operator measured all nine samples and was blinded to the results of the other operators. The overall percent deviation was 4.3%, which is relatively low and was considered the maximal accuracy that could be expected of an inline OD model.

Next, a series of 15 fermentation runs were completed during normal process development at Sanofi, where the PIMS collected raw transmission and reflection data from the OD probes and stored offline OD measurements to serve as reference points. Completing 15 fermentation runs is not a minimum requirement; however, it did provide a complex and realistic dataset comprising five different OD probes, five different proteins of interest expressed, two different chemically‐defined media, and two different antifoams used. The common feature of these fermentation experiments was the use of the same *E. coli* strain and chemically defined media. We included five different recombinant protein targets (all intracellularly expressed, soluble, and non‐toxic to the host) to evaluate whether the multivariate model is transferable across projects that share a common microbe, regardless of the specific project or protein expressed. Secondly, although the chemically‐defined media and antifoams may display subtle differences due to changes in raw material sourcing, these differences are unlikely to have a major impact. Nevertheless, for transparency reasons, we classified and compared the data based on material sources (see below).

While evaluating the initial dataset, it was immediately apparent that the raw transmission and reflection dataset required smoothing due to significant fluctuations in signal intensity (Figure 1b). A moving average (MA) with a 0.2 h window was selected as it was the minimum duration that produced a smooth line during the “waiting phase” (Figure 1b,w), where the fermenter is equilibrating (aerating, heating, and stirring) but has not yet been inoculated.

Intuitively, the transmission signal plays a more important role during low‐density *E. coli* culture, as the reflection signal is low. In contrast, the reflection signal plays a more important role during higher‐density *E. coli* culture, such as during fed‐batch and induction (IN) phases (Figure 1b). Additional data transformation was required for transmission, as the MA was non‐linearly correlated to OD offline (Figure 1c), as was a log transformation (Figure 1d). However, the inverse of the transmission MA produced a seemingly linear relationship with offline OD (Figure 1e). Reflection MA also seemingly had a linear relationship with offline OD (Figure 1f).

While building the dataset of 15 fermenter runs and their associated offline samples, it was suspected that antifoam addition could potentially affect an inline OD probe given that the stock bottle of antifoam used during fermentation is a solution that appears nearly opaque. An OD measurement of antifoam is difficult to accurately and consistently report since the OD of the oil‐in‐water emulsion is sensitive to dilution in water (e.g., 1.5 OD with 1:6 dilution, and 15 OD with 1:50 dilution). Therefore, the PIMS tracked the volume of diluted antifoam addition as a percentage of total fermenter volume (termed “antifoam (%vol)”), which also appeared correlated with offline OD (Figure 1g). As the 15 runs were operated differently, common predictors such as fermenter volume were not suitable for predicting OD (Figure 1h). Parameters with reasonable linear correlations with offline OD were included in subsequent multivariate analysis.

**FIGURE 2:**
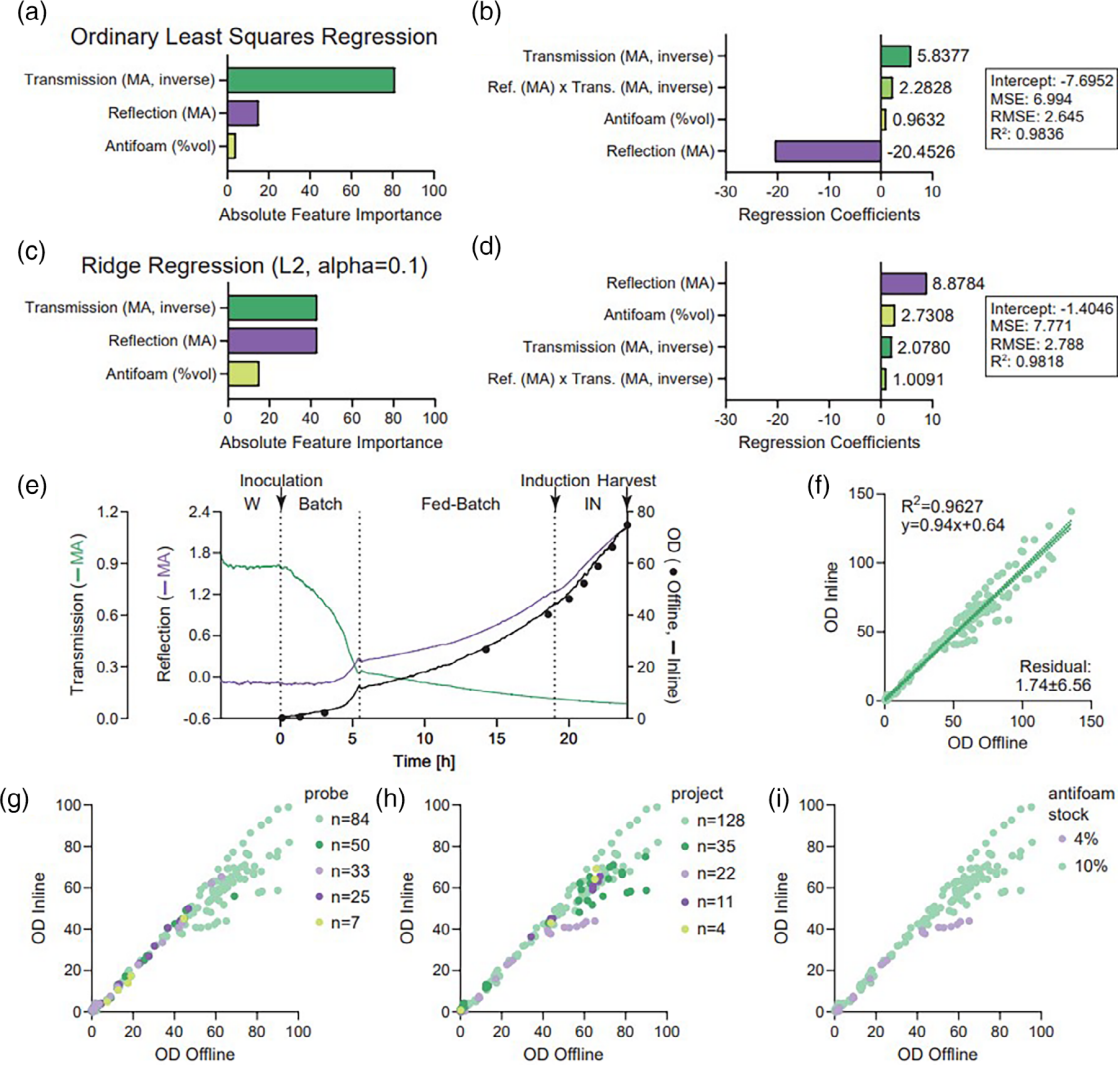
Multivariate model development for inline OD model. (**a**) Absolute feature importance for ordinary least squares regression (OLSR). (**b**) Regression coefficients, intercept, and additional model metrics for OLSR. (**c**) Absolute feature importance for Ridge regression. (**d**) Regression coefficients, intercept, and additional model metrics for Ridge regression. (**e**) Representative MA continuous data from OD probe collected during waiting (W), batch, fed‐batch, and induction (IN) phases, together with the multivariate equation generated using OLSR regression coefficients and intercept. OD offline included for comparison. (**f**) Comparison of inline vs. offline OD for all *N* = 15 fermenter runs and *N* = 159 offline samples collected. (**g‐i**) Visualization of inline vs. offline OD, subcategorized by five different probes (**g**), five different projects (**h**), and two different stock concentrations of antifoams used (**i**).

Altogether, the data from these aggregated fermentation runs were used to build a multivariate model in Dataiku, where ordinary least squares regression (OLSR) and Ridge regression were compared. OLSR found that transmission (MA, inverse) was by far the most important feature, followed by reflection (MA) and antifoam (%vol) (Figure 2a). Feature interactions between transmission and reflection were also observed and contributed to the regression coefficients (Figure 2b). The OLSR model accuracy was high, with a root mean square error (RMSE) of 2.6 and *R* ^2^ = 0.9840. Next, ridge regression was performed on the same dataset, which led to near‐equal importance of transmission (MA, inverse) and reflection (MA), and an elevated relative importance of antifoam (%vol) (Figure 2c). Despite these differences and the difference in regression coefficients, both models performed similarly. The Ridge regression model accuracy was also high, with a RMSE of 2.7 and *R* ^2^ = 0.9820 (Figure 2c), with similar feature interactions between reflection and transmission (Figure 2d) as observed for OLSR (Figure 2b). However, it was unexpected for the antifoam (%vol) feature to play such a significant role in the regression coefficients (Figure 2d). While either model would produce useful inline OD results, OLSR was selected as it was marginally more accurate and placed less weight on the antifoam (%vol) feature.

An example of a successful fermentation run with inline OD calculated using the PIMS software in real‐time is shown in Figure 2e, using the OLSR regression coefficients. The waiting phase (W), where the fermenter is equilibrating, produces flat lines for the MA of the raw transmission and reflection data, and inline OD is calculated as zero. Upon inoculation, a batch and fed‐batch phase ensue. Offline data could not be captured between 3 and 14 hours as this portion of the run occurred overnight; however, the inline OD was able to “fill in the gaps” to provide continuous coverage. Overall model performance appeared good for this single run (Figure 2e), as well as an aggregation of all 15 runs (*n* = 159 offline datapoints; Figure 2f) over a large dynamic range from 0 to 140 OD with high density *E. coli* culture. Upon closer inspection, there appeared to be no substantial variations for inline OD prediction capability due to the five different OD probes (Figure 2g), five different proteins of interest expressed (Figure 2h), or two different antifoams concentrations used (Figure 2i).

In a fast‐paced process development platform, it is highly desirable to have a global PIMS‐based inline OD multivariate model as opposed to training new OD probes and having each probe use its own model. This reduces workload and prevents the need to track potential batch‐to‐batch variations across probes and models. However, minor differences between probes are expected, which likely account for some model variability. When compared to the original offline variability of 4.3% at an OD of 80.8 (Figure 1a), which represents a range of 77.4 to 84.2 (width: 6.8 OD), the OLSR model predicts a reading with an average residual of 1.74 ± 6.56 (Figure 2f). Therefore, for an offline reading of 80.8, this would likely produce an inline OD reading within 75.9 to 89.1. This prediction range (width: 13.2) was considered acceptable, since operator variability accounted for 6.8 OD of this range.

**FIGURE 3:**
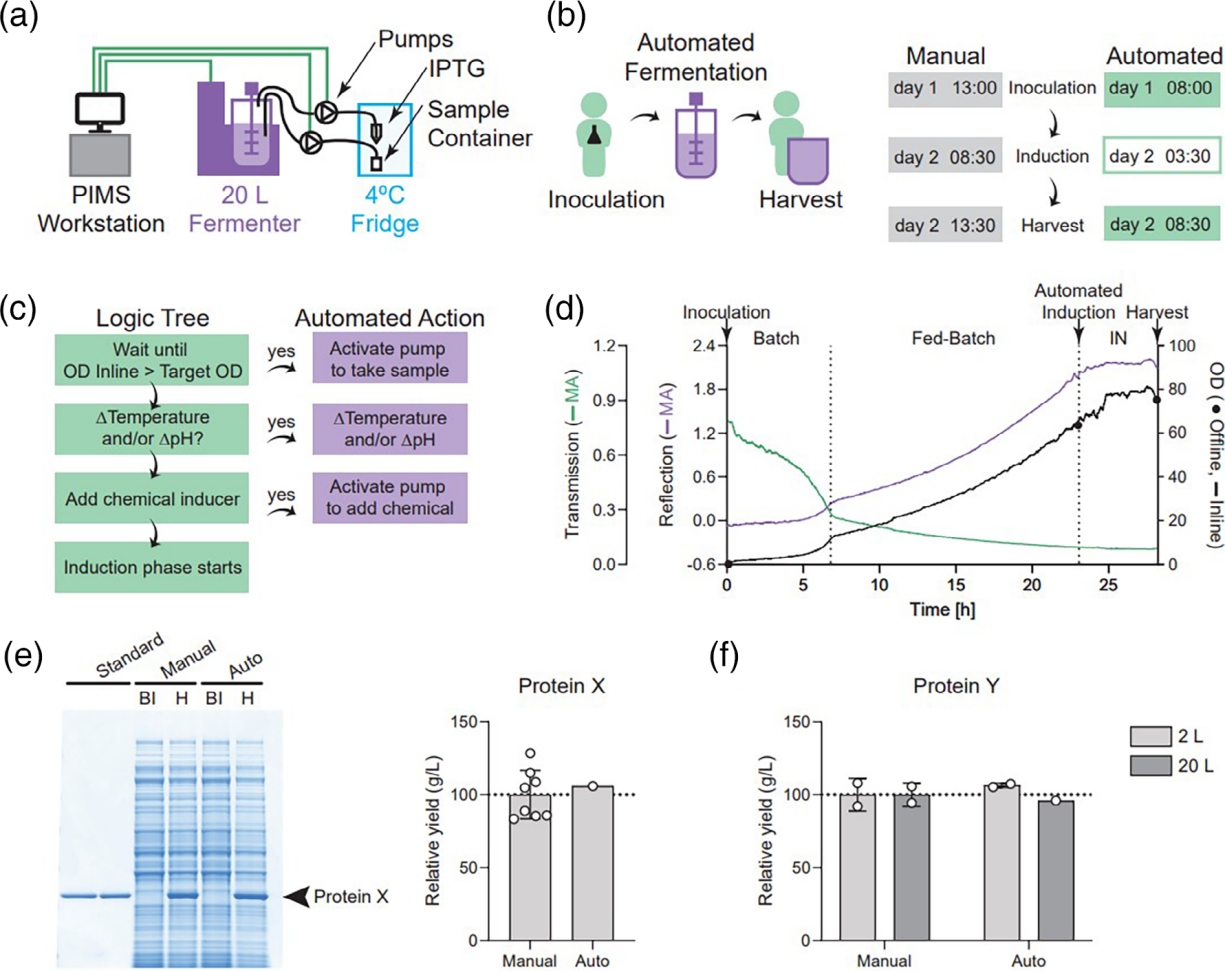
Technical transfer demonstration of inline OD model at pilot scale. (**a**) Diagram of PIMS and accessory connections to the 20 L fermenter (in purple). Green lines represent digital communications, and black lines represent sterile tubing connections. (**b**) Representative depiction of minimal manual interventions required during automated fermentation. When the initiation of the induction phase is automated, the timing of fermentation is more flexible and can be tailored to maximize production hours. (**c**) Summary of the logic tree existing in PIMS to automate the steps required for the initiation of the induction phase. (**d**) Representative MA continuous data from the OD probe collected in the 20 L fermenter with the new (120 mm) OD probe during batch, fed‐batch, and induction (IN) phases. OD offline included for comparison. The sample taken upon the initiation of the induction phase was taken automatically by a pump controlled by PIMS and stored at 4°C until the following morning, when it could be measured offline. (**e**) Representative SDS‐PAGE showing before induction (BI) and harvest (H) total cell lysate from two 2‐L fermentation runs with equivalent operating conditions in parallel to produce Protein X. Yield normalized to the average of manual yield; no statistics performed due to sample size. (**f**) Relative yields from harvest total cell lysate from 2‐L and 20‐L fermentations run with equivalent operating conditions and assessing Protein X yield (yield normalized to average of manual yield). Two‐way ANOVA, no significant differences.

The significant value of an inline OD probe rests on the application of automating the initiation of induction. As previously mentioned, having a PIMS‐based multivariate model allows for model transfer and automation of events triggering the induction phase. To mimic a transferred process, a 20 L pilot fermentation run was completed with a new 120 mm OD probe (since the 2‐L fermenters use a longer 220 mm OD probe, which was used for model building) (Figure 3a). Automating the induction phase would reduce the number of manual operations with the fermenter down to only two: inoculation and harvest (Figure 3b). Another benefit from this automation is no longer needing to coordinate the start of the induction phase during regular working hours (Figure 3b, manual). Alternatively, when the induction phase is automatically initiated, only the inoculation and harvest timepoints need to occur during regular working hours, offering more flexibility in scheduling (Figure 3b, automated).

The final component in preparation of the “transferred pilot process” was designing the logic tree in the PIMS software, which is summarized in Figure 3c. The PIMS would wait until the inline OD exceeded the target OD (Figure 3c), collect a culture sample, adjust culture conditions if necessary, and then add the induction agent. As observed in the trend chart over the course of the “transferred process” run, the transmission and reflection MA were calculated (Figure 3d), and the data were similar to the previous 2‐L studies (Figure 2e). Furthermore, a fully automated sample was drawn at the start of the induction phase and stored appropriately until it could be measured on the following day. The two manual offline OD measurements performed for the inoculation and harvest time points were very close to the inline OD values. Subsequent 20‐L pilot runs were successfully executed with accurate automated induction phase initiation, where inline OD data was similar to offline measurements.

To ensure consistent process yields, we directly compared the relative yields from manual versus automated induction fermentations for two different recombinant protein targets: Protein X at 2‐L scale (Figure 3e) and Protein Y at both the 2‐L and 20‐L scales (Figure 3f; two‐way ANOVA, no significant differences), where all operating parameters were otherwise equivalent between the manual and automated induction runs. Furthermore, we did not observe any significant differences in overall protein expression as assessed by SDS‐PAGE when comparing the two induction strategies (Figure 3e), noting these two runs were performed in parallel to control for any potential batch effects. Altogether, automating the induction initiation was successful using a transferable multivariate model for a new OD probe without the need to develop a new offline correlation model prior to use. Other advantages include rich continuous data capture, workload reduction, and minimization of contamination risks from frequent manual sampling.

## 4 DISCUSSION

As industries seek efficiency, sustainability, and enhanced performance, many bioprocesses have undergone transformation using “Industry 4.0” principles.^16^ Automation and digitalization have and continue to revolutionize traditional manufacturing processes, and here we show the implementation of PAT with a PIMS to automate the “final step” of fermentation: the induction phase. Initial benefits of this solution include greater flexibility in operational scheduling, reduced workload, and continuous data capture. Relying on an offline measurement to trigger the induction phase may also require a larger range in acceptable values to prevent out‐of‐specification results. Therefore, an inline OD probe and automated induction would enable smaller variation in OD and likely have a positive effect on batch‐to‐batch consistency. We also demonstrate the transfer of a multivariate model from bench scale to lab scale, without the need to generate a new offline correlation model for each new OD probe. This maximizes efficiency and has a significant impact in global companies and other industries.

These applications are not limited to the technologies used in this study. The authors present transmission and reflection data recorded from Dencytee probes. However, comparable and competitive alternatives exist, including capacitance and other turbidity approaches. Capacitance probes are generally reserved for cell culture as the dielectric technology can provide greater detail during infection processes and inform on live biomass^17^; however, they have been used to monitor *E. coli* cultures as well.^18^ Additional turbidity approaches, such as a function of near infrared absorbance, or Optek turbidity probes with “concentration units” may require application‐specific calibration or offline correlation models. The authors are not aware of reports where a standard “out‐of‐the‐box” DCS has been used to determine a set inline OD threshold and then activate a pump to add a chemical induction agent (or change the fermenter temperature set point for thermal induction). However, it may be possible to customize DCS systems to enable more complex feedback control loops. Alternatively, a PIMS and/or data management system solution may be required to co‐exist with industrial DCS setups. The authors are not aware of software solutions equivalent to the automation capability achieved by Securecell's Lucullus; however, additional PIMS and/or data management systems that may also be considered include SynTQ (Optimal Industrial Technologies Ltd), SIMATIC SIPAT (Siemens AG), xPAT (ABB), and LabVIEW (National Instruments Corp),^19^ each with their own inherent benefits and drawbacks that may be application‐specific. The present study found that the digital solutions used were not cost‐prohibitive; however, initial setup requires investment and training.

The transfer of models trained on offline OD measurements includes inherent considerations to ensure precision and accuracy. Classical reports identified many of these “transferability” considerations. An OD prediction equation, such as the one presented in this study for *E. coli*, may not be suitable for other cellular suspensions that have a different extinction coefficient,^20^ especially some *E. coli* strains that may change cell shape via filamentation during times of cell stress.^21^ It is possible that cell stress could occur during poorly controlled fermentation processes as well as induction, suggesting that the inline OD prediction model may be less accurate during these scenarios. However, suppression of cell filamentation is possible with well‐controlled processes as well as optimization of the induction parameters. Potential changes in growth rates during induction,^22^ can also be identified and monitored using an inline OD prediction model to allow this optimization process to occur more efficiently. Furthermore, the Beer–Lambert Law maintains a linear relationship between the raw spectrophotometer reading and the concentration of particles only when measuring dilute solutions (i.e., diluted *E. coli* samples). These raw spectrophotometer readings may also differ across instruments, and even due to apertures, slit widths, and wavelengths used within one instrument.^23^ In addition, variation in measurements among operators may also be expected. This can be evaluated with technical replicates as completed in Figure 1a, to determine an acceptable level of precision. Lastly, all OD measurements in the present study are based on *E. coli* cultured in chemically defined media. It is possible that undefined media, which can be cloudy in appearance, may lead to an inline OD model that is different from the present solution. Despite these technical limitations, alignment of practices is not an insurmountable challenge and can be achieved across global industrial sites.

## 5 CONCLUSIONS

Beyond the automation of the induction phase, similar paradigms can be used in multiple additional industries. These applications include monitoring the separation of solids during centrifugation control, and in the pulp and paper industry where white liquor and/or black liquor are monitored during the causticizing process to remove particulates and lime mud.^24^ Other industrial processes may use PAT to monitor and control OD including, for instance, brewhouses' brewing yeast and management of turbidity, palm oil mill management of mixed liquor suspended solids,^25^ and other food and beverage production and filtration applications at the industrial scale.

Future directions include using the inline OD multivariate model to also predict harvest wet cell weight and/or dry cell weight. Harvest wet cell weight is important in biopharmaceutical manufacturing for subsequent microfiltration and centrifugation processes where parameters such as kilograms of biomass per membrane area (kg/m^2^) are factored. The future benefits of contextualized data and automation capacity from PIMS‐based control of bioreactors include model predictive control and process modeling applications consistent with Industry 4.0 standards.

## AUTHOR CONTRIBUTIONS

All authors substantially contributed to the conception or design of the work; or the acquisition, analysis, or interpretation of data for the work, and provided final approval of the version to be published. J.R., A.S., and J.N. contributed to drafting the work and revising it critically for important intellectual content.

## FUNDING INFORMATION

This study was funded by Sanofi Vaccines.

## CONFLICT OF INTEREST STATEMENT

All authors are current and/or former employees of Sanofi and may hold shares and/or stock options in the company.

## Data Availability

The data that support the findings of this study are available from the corresponding author upon reasonable request.
